# Verifying Array Manipulating Programs with Full-Program Induction

**DOI:** 10.1007/978-3-030-45190-5_2

**Published:** 2020-03-13

**Authors:** Supratik Chakraborty, Ashutosh Gupta, Divyesh Unadkat

**Affiliations:** 8 Johannes Kepler University, Linz, Austria; 9 University of Birmingham, Birmingham, UK; 10 Indian Institute of Technology Bombay, Mumbai, India; 11 TCS Research, Pune, India

## Abstract

We present a full-program induction technique for proving (a sub-class of) quantified as well as quantifier-free properties of programs manipulating arrays of parametric size *N*. Instead of inducting over individual loops, our technique inducts over the entire program (possibly containing multiple loops) directly via the program parameter *N*. Significantly, this does not require generation or use of loop-specific invariants. We have developed a prototype tool Vajra to assess the efficacy of our technique. We demonstrate the performance of Vajra vis-a-vis several state-of-the-art tools on a set of array manipulating benchmarks.

## 1 Introduction

Programs with loops manipulating arrays are common in a variety of applications. Unfortunately, assertion checking in such programs is undecidable. Existing tools therefore use a combination of techniques that work well for certain classes of programs and assertions, and yield conservative results otherwise. In this paper, we present a new technique to add to this arsenal of techniques. Specifically, we focus on programs with loops manipulating arrays, where the size of each array is a symbolic integer parameter $N$ ($> 0$). We allow (a sub-class of) quantified and quantifier-free pre- and post-conditions that may depend on the symbolic parameter $N$. Thus, the problem we wish to solve can be viewed as checking the validity of a parameterized Hoare triple $\{\varphi(N)\}\ \mathsf{P}_N\ \{\psi(N)\}$ for all values of $N$ ($> 0$), where the program $\mathsf{P}_N$ computes with arrays of size $N$, and $N$ is a free variable in $\varphi(\cdot)$ and $\psi(\cdot)$. Fig. 1(a) shows an example of one such Hoare triple, written using `assume` and `assert`. This triple effectively verifies that $\sum_{j=0}^{i-1}\left(1+\sum_{k=0}^{j-1} 6\cdot(k+1)\right) = i^3$ for all $i \in \{0 \ldots N-1\}$, and for all $N > 0$. Although each loop in Fig. 1(a) is simple, their sequential composition makes it difficult even for state-of-the-art tools like VIAP [26], VeriAbs [8], FreqHorn [10], Tiler [4], Vaphor [24], or Booster [1] to prove the post-condition correct. In fact, none of the above tools succeed in automatically proving the post-condition in Fig. 1(a). In contrast, the technique presented in this paper, called *full-program induction*, proves the post-condition in Fig. 1(a) correct within a few seconds.

Like several earlier approaches [29], full-program induction relies on mathematical induction to reason about programs with loops. However, the way in

```
// assume(true)
1. for (int t1=0; t1<N; t1=t1+1) {
2.   if (t1==0) { A[t1] = 6; }
3.   else { A[t1] = A[t1-1]+6; }
4. }
5. for (int t2=0; t2<N; t2=t2+1) {
6.   if (t2==0) { B[t2] = 1; }
7.   else { B[t2] = B[t2-1]+A[t2-1]; }
8. }
9. for (int t3=0; t3<N; t3=t3+1) {
10.  if (t3==0) { C[t3] = 0; }
11.  else { C[t3] = C[t3-1]+B[t3-1]; }
12.}
// assert(forall i in 0..N-1, C[i]= i^3)
```

(a)

```
// assume(true)
   A[0] = 6;
   B[0] = 1;
   C[0] = 0;
// assert((C[0] = 0^3) and (B[0] = 1^3 - 0^3) and
//        (A[0] = 2^3 - 2*1^3 + 0^3))
```

(b)

```
// assume((N > 1) and (C_Nm1[N-2] = (N-2)^3) and
//        (B_Nm1[N-2] = (N-1)^3 - (N-2)^3) and
//        (A_Nm1[N-2] = N^3 - 2*(N-1)^3 + (N-2)^3))
   A[N-1] = A_Nm1[N-2] + 6;
   B[N-1] = B_Nm1[N-2] + A_Nm1[N-2];
   C[N-1] = C_Nm1[N-2] + B_Nm1[N-2];
// assert((C[N-1] = (N-1)^3) and
//        (B[N-1] = N^3 - (N-1)^3) and
//        (A[N-1] = (N+1)^3 - 2*N^3 + (N-1)^3))
```

(c)

**Fig. 1.** Original and simplified Hoare triples which the inductive claim is formulated and proved differs significantly. Specifically, (i) we *do not require explicit or implicit loop-specific invariants* to be provided by the user or generated by a solver (viz. by constrained Horn clause solvers [21,15,10] or recurrence solvers [26,17]), (ii) we *induct on the full program* (possibly containing multiple loops) with parameter $N$ and not on iterations of individual loops in the program, and (iii) we perform *non-trivial correct-by-construction code transformations*, whenever feasible, to simplify the inductive step of reasoning. The combination of these factors often reduces reasoning about a program with multiple loops to reasoning about one with fewer (sometimes even none) and "simpler" loops, thereby simplifying proof goals. In this paper, we demonstrate this, focusing on programs with sequentially composed, but non-nested loops.

As an illustration of simplifications that can result from application of full-program induction, consider the problem in Fig. 1(a) again. Full-program induction reduces checking the validity of the Hoare triple in Fig. 1(a) to checking the validity of two "simpler" Hoare triples, represented in Figs. 1(b) and 1(c). Note that the programs in Figs. 1(b) and 1(c) are loop-free. In addition, their pre- and post-conditions are quantifier-free. The validity of these Hoare triples (Figs. 1(b) and 1(c)) can therefore be easily proved, e.g. by bounded model checking [6] with a back-end SMT solver like Z3 [25]. Note that the value computed in each iteration of each loop in Fig. 1(a) is data-dependent on previous iterations of the respective loops. Hence, none of these loops can be trivially translated to a set of parallel assignments.

Invariant-based techniques, viz. [13,16,23,7,14,30,2,19], are popularly used to reason about array manipulating programs. If we were to prove the assertion in Fig. 1(a) using such techniques, it would be necessary to use appropriate loop-specific invariants for each of the three loops in Fig. 1(a). The weakest loop invariants needed to prove the post-condition in this example are: $\forall i \in [0...t1-1]\ (A[i] = 6i+6)$ for the first loop (lines 1-4), $\forall j \in [0...t2-1]\ (B[j] = 3j^2+3j+1) \wedge (A[j] = 6j+6)$ for the second loop (lines 5-8), and $\forall k \in [0...t3-1]\ (C[k] = k^3) \wedge (B[k] = 3k^2+3k+1)$ for the third loop (lines

9-12). Unfortunately, automatically deriving such quantified non-linear loop invariants is far from trivial. Template-based invariant generators, viz. [12,9], are among the best-performers when generating such complex invariants. However, their abilities are fundamentally limited by the set of templates from which they choose. We therefore choose not to depend on invariants for individual loops in our work at all. Instead of inducting over the iterations of each individual loop, we propose to reason about the entire program (containing one or more loops) directly, while inducting on the parameter $N$. Needless to say, each approach has its own strengths and limitations, and the right choice always depends on the problem at hand. Our experiments show that full-program induction is able to solve several difficult problem instances with an off-the-shelf SMT solver (Z3) at the back-end, which other techniques either fail to solve these instances, or rely on sophisticated recurrence solvers.

The primary contributions of our work can be summarized as follows.

- We introduce the notion of *full-program induction* for reasoning about assertions in programs with loops manipulating arrays.
- We present practical algorithms for full-program induction.
- We describe a prototype tool Vajra that implements the algorithms, using an off-the-shelf SMT solver, viz. Z3, at the back-end to discharge verification conditions. Vajra outperforms several state-of-the-art tools on a suite of array-manipulating benchmark programs.

***Related Work.*** Earlier work on inductive techniques can be broadly categorized into those that require loop-specific invariants to be provided or automatically generated, and those that work without them. Requiring a "good" inductive invariant for every loop in a program effectively shifts the onus of assertion checking to that of invariant generation. Among techniques that do not require explicit inductive invariants or mid-conditions for each loop, there are some that require loop invariants to be implicitly generated by a constraint solver. These include techniques based on constrained Horn clause solving [21,15,10,24], acceleration and lazy interpolation for arrays [1] and those that use inductively defined predicates and recurrence solving [26,17], among others. Thanks to the impressive capabilities of modern constraint solvers and the effectiveness of carefully tuned heuristics for stringing together multiple solvers, this approach has shown a lot of promise in recent years. However, at a fundamental level, these formulations rely on solving implicitly specified loop invariants garbed as constraint solving problems. There are yet other techniques, such as that in [28], that truly do not depend on loop invariants being generated. In fact, the technique of [28] comes closest to our work in principle. However, [28] imposes severe restrictions on the input programs, and the example in Fig. 1 does not meet these restrictions. Therefore, the technique of [28] is applicable only to a small part of the program-assertion space over which our technique works. Techniques such as tiling [4] reason one loop at a time and apply only when loops have simple data dependencies across iterations (called *non-interference* of tiles in [4]). It effectively uses a slice of the post-condition of a loop as an inductive invariant, and also requires strong enough mid-conditions to be generated in the case of sequentially composed loops. We circumvent all of these requirements in the current work. For some other techniques for analyzing array manipulating programs, please see [7,19,18].

## 2 Overview of Full-program Induction

Recall that our objective is to check the validity of the parameterized Hoare triple $\{\varphi(N)\}\ \mathsf{P}_N\ \{\psi(N)\}$ for all $N > 0$. At a high level, our approach works like any other inductive technique. Thus, we have a base case, where we verify that the parameterized Hoare triple holds for some small values of $N$, say $0 < N \leq M$. We then hypothesize that $\{\varphi(N-1)\}\ \mathsf{P}_{N-1}\ \{\psi(N-1)\}$ holds for some $N > M$, and try to show that this implies $\{\varphi(N)\}\ \mathsf{P}_N\ \{\psi(N)\}$. While this sounds simple in principle, there are several technical difficulties en route. Our contribution lies in overcoming these difficulties algorithmically for a large class of programs and assertions, thereby making *full-program induction* a viable and competitive technique for proving properties of array manipulating programs.

We rely on an important, yet reasonable, assumption that can be stated as follows: *For every value of $N$ $(> 0)$, every loop in $\mathsf{P}_N$ can be statically unrolled a fixed number (say $f(N)$) of times to yield a loop-free program $\widehat{\mathsf{P}_N}$ that is semantically equivalent to $\mathsf{P}_N$.* Note that this does not imply that reasoning about loops can be translated into loop-free reasoning. In general, $f(N)$ is a non-constant function, and hence, the number of unrollings of loops in $\mathsf{P}_N$ may strongly depend on $N$. In our experience, loops in a vast majority of array manipulating programs (including Fig. 1(a)) satisfy the above assumption. Consequently, the base case of our induction reduces to checking a Hoare triple for a loop-free program. Checking such a Hoare triple is easily achieved by compiling the pre-condition, program and post-condition into an SMT formula, whose (un)satisfiability can be checked with an off-the-shelf back-end SMT solver.

The inductive step is the most complex one, and is the focus of the rest of the paper. Recall that the inductive hypothesis asserts that $\{\varphi(N-1)\}\ \mathsf{P}_{N-1}\ \{\psi(N-1)\}$ is valid. To make use of this hypothesis in the inductive step, we must relate the validity of $\{\varphi(N)\}\ \mathsf{P}_N\ \{\psi(N)\}$ to that of $\{\varphi(N-1)\}\ \mathsf{P}_{N-1}\ \{\psi(N-1)\}$. We propose doing this, whenever possible, via two key notions – that of "difference" program and "difference" pre-condition. Given a parameterized program $\mathsf{P}_N$, intuitively the "difference" program $\partial\mathsf{P}_N$ is one such that $\mathsf{P}_{N-1};\partial\mathsf{P}_N$ is semantically equivalent to $\mathsf{P}_N$, where ";" denotes sequential composition. It turns out that for our purposes, the semantic equivalence alluded to above is not really necessary; it suffices to have $\partial\mathsf{P}_N$ such that $\{\varphi(N)\}\ \mathsf{P}_N\ \{\psi(N)\}$ is valid iff $\{\varphi(N)\}\ \mathsf{P}_{N-1};\partial\mathsf{P}_N\ \{\psi(N)\}$ is valid. We will henceforth use this interpretation of a "difference" program. The "difference" pre-condition $\partial\varphi(N)$ is a formula such that (i) $\varphi(N) \rightarrow (\varphi(N-1) \wedge \partial\varphi(N))$ and (ii) the execution of $\mathsf{P}_{N-1}$ doesn't affect the truth of $\partial\varphi(N)$. Computing $\partial\mathsf{P}_N$ and $\partial\varphi(N)$ is not easy in general, and we discuss this in detail in the rest of the paper.

Assuming we have $\partial\mathsf{P}_N$ and $\partial\varphi(N)$ with the properties stated above, the proof obligation $\{\varphi(N)\}\ \mathsf{P}_N\ \{\psi(N)\}$ can now be reduced to proving $\{\varphi(N-$

$1)\}$ $\mathsf{P}_{N-1}$ $\{\psi(N-1)\}$ and $\{\psi(N-1) \wedge \partial\varphi(N)\}$ $\partial\mathsf{P}_N$ $\{\psi(N)\}$. The first triple follows from the inductive hypothesis. Proving the second triple may require strengthening the pre-condition, say by a formula $\mathsf{Pre}(N-1)$, in general. Recalling that we are in the inductive step of mathematical induction, we formulate the new proof sub-goal in such a case as $\{(\psi(N-1) \wedge \mathsf{Pre}(N-1)) \wedge \partial\varphi(N)\}$ $\partial\mathsf{P}_N$ $\{\psi(N) \wedge \mathsf{Pre}(N)\}$. While this is somewhat reminiscent of loop invariants, observe that $\mathsf{Pre}(N)$ is *not* really a loop-specific invariant. Instead, it is analogous to computing an invariant for the entire program, possibly containing multiple loops. Specifically, the above process strengthens both the pre- and post-condition of $\{\psi(N-1) \wedge \partial\varphi(N)\}$ $\partial\mathsf{P}_N$ $\{\psi(N)\}$ simultaneously using $\mathsf{Pre}(N-1)$ and $\mathsf{Pre}(N)$, respectively. The strengthened post-condition of the resulting Hoare triple may, in turn, require a new pre-condition $\mathsf{Pre}'(N-1)$ to be satisfied. This process of strengthening the pre- and post-conditions of the Hoare triple involving $\partial\mathsf{P}_N$ can be iterated until a fix-point is reached, i.e. no further pre-conditions are needed for the parameterized Hoare triple to hold. While the fix-point was quickly reached for all benchmarks we experimented with, we also discuss how to handle cases where the above process may not converge easily. Note that since we effectively strengthen the pre-condition of the Hoare triple in the inductive step, for the overall induction to go through, it is also necessary to check that the strengthened assertions hold at the end of each base case check. The technique described above is called *full-program induction*, and the following theorem guarantees its soundness.

**Theorem 1.** *Given* $\{\varphi(N)\}$ $\mathsf{P}_N$ $\{\psi(N)\}$*, suppose the following are true:*

1. *For* $N > 1$, $\{\varphi(N)\}$ $\mathsf{P}_{N-1};\partial\mathsf{P}_N$ $\{\psi(N)\}$ *holds iff* $\{\varphi(N)\}$ $\mathsf{P}_N$ $\{\psi(N)\}$ *holds.*
2. *For* $N > 1$, *there exists a formula* $\partial\varphi(N)$ *such that (a)* $\partial\varphi(N)$ *doesn't refer to any program variable or array element modified in* $\mathsf{P}_{N-1}$*, and (b)* $\varphi(N) \rightarrow \varphi(N-1) \wedge \partial\varphi(N)$.
3. *There exists an integer* $M \geq 1$ *and a parameterized formula* $\mathsf{Pre}(M)$ *such that (a)* $\{\varphi(N)\}$ $\mathsf{P}_N$ $\{\psi(N)\}$ *holds for* $0 < N \leq M$*, (b)* $\{\varphi(M)\}$ $\mathsf{P}_M$ $\{\psi(M) \wedge \mathsf{Pre}(M)\}$ *holds, and (c)* $\{\psi(N-1) \wedge \mathsf{Pre}(N-1) \wedge \partial\varphi(N)\}$ $\partial\mathsf{P}_N$ $\{\psi(N) \wedge \mathsf{Pre}(N)\}$ *holds for* $N > M$.

*Then* $\{\varphi_N\}$ $\mathsf{P}_N$ $\{\psi_N\}$ *holds for all* $N \geq 1$.

*Proof.* For $0 < N \leq M$, condition 3(a) ensures that $\{\varphi(N)\}$ $\mathsf{P}_N$ $\{\psi(N)\}$ holds. For $N > M$, note that by virtue of condition 1 and 2(b), $\{\varphi(N)\}$ $\mathsf{P}_N$ $\{\psi(N)\}$ holds if $\{\varphi(N-1) \wedge \partial\varphi(N)\}$ $\mathsf{P}_{N-1};\partial\mathsf{P}_N$ $\{\psi(N) \wedge \mathsf{Pre}(N)\}$ holds. With $\psi(N-1) \wedge \mathsf{Pre}(N-1)$ as a mid-condition, and by virtue of condition 2(a), the latter Hoare triple holds for $N > M$ if $\{\varphi(M)\}$ $\mathsf{P}_M$ $\{\psi(M) \wedge \mathsf{Pre}(M)\}$ holds and $\{\psi(N-1) \wedge \mathsf{Pre}(N-1) \wedge \partial\varphi(N)\}$ $\partial\mathsf{P}_N$ $\{\psi(N) \wedge \mathsf{Pre}(N)\}$ holds for all $N > M$. Both these triples are seen to hold by virtue of conditions 3(b) and (c). □

## 3 Algorithms for Full-program Induction

We now discuss the *full-program induction* algorithm, focusing on generation of three crucial components: *difference program* $\partial\mathsf{P}_N$, *difference pre-condition* $\partial\varphi(N)$, and the formula $\mathsf{Pre}(N)$ for strengthening pre- and post-conditions.

### 3.1 Preliminaries

We consider array manipulating programs generated by the grammar shown below (adapted from [4]).

$$
\begin{aligned}
\mathsf{PB} &::= \mathsf{St} \\
\mathsf{St} &::= v := \mathsf{E} \mid A[\mathsf{E}] := \mathsf{E} \mid \mathbf{if}(\mathsf{BoolE})\ \mathbf{then}\ \mathsf{St}\ \mathbf{else}\ \mathsf{St} \mid \mathsf{St}\ ;\ \mathsf{St} \mid \\
&\quad\ \mathbf{for}\ (\ell := 0;\ \ell < \mathsf{E};\ \ell := \ell{+}1)\ \{\mathsf{St1}\} \\
\mathsf{St1} &::= v := \mathsf{E} \mid A[\mathsf{E}] := \mathsf{E} \mid \mathbf{if}(\mathsf{BoolE})\ \mathbf{then}\ \mathsf{St1}\ \mathbf{else}\ \mathsf{St1} \mid \mathsf{St1}\ ;\ \mathsf{St1} \\
\mathsf{E} &::= \mathsf{E}\ \mathsf{op}\ \mathsf{E} \mid A[\mathsf{E}] \mid v \mid \ell \mid \mathsf{c} \mid N \\
\mathsf{op} &::= + \mid \text{-} \mid * \mid / \\
\mathsf{BoolE} &::= \mathsf{E}\ \mathsf{relop}\ \mathsf{E} \mid \mathsf{BoolE}\ \mathsf{AND}\ \mathsf{BoolE} \mid \mathsf{NOT}\ \mathsf{BoolE} \mid \mathsf{BoolE}\ \mathsf{OR}\ \mathsf{BoolE}
\end{aligned}
$$

This grammar restricts programs to have non-nested loops. While this limits the set of programs to which our technique currently applies, there is a large class of useful programs, with possibly long sequences of loops, that are included in the scope of our work. In reality, our technique also applies to a subclass of programs with nested loops. However, characterizing this class of programs through a grammar is a bit unwieldy, and we avoid doing so for reasons of clarity. A program $\mathsf{P}_N$ is a tuple $(\mathcal{V}, \mathcal{L}, \mathcal{A}, \mathsf{PB}, N)$, where $\mathcal{V}$ is a set of scalar variables, $\mathcal{L} \subseteq \mathcal{V}$ is a set of scalar loop counter variables, $\mathcal{A}$ is a set of array variables, $\mathsf{PB}$ is the program body, and $N$ is a special symbol denoting a positive integer parameter. In the grammar shown above, we assume $A \in \mathcal{A}$, $v \in \mathcal{V} \setminus \mathcal{L}$, $\ell \in \mathcal{L}$ and $\mathsf{c} \in \mathbb{Z}$. Furthermore, "relop" is assumed to be one of the relational operators and "op" is an arithmetic operator from the set {+, -, *, /}. We also assume that each loop $L$ has a unique loop counter variable $\ell$ which is initialized at the beginning of $L$ and is incremented by 1 at the end of each iteration. Assignments in the body of $L$ are assumed not to update $\ell$. Finally, for each loop with termination condition $\ell < \mathsf{E}$, we assume that $\mathsf{E}$ is an expression in terms of $N$. We denote by $k_L(N)$ the number of times loop $L$ iterates in the program with parameter $N$. We verify Hoare triples of the form $\{\varphi(N)\}\ \mathsf{P}_N\ \{\psi(N)\}$, where $\varphi(N)$ and $\psi(N)$ are either universally quantified formulas of the form $\forall I\ (\Phi(I, N) \implies \Psi(\mathcal{A}, \mathcal{V}, I, N))$ or quantifier-free formulas of the form $\Xi(\mathcal{A}, \mathcal{V}, N)$. In the above, $I$ is a sequence of array index variables, $\Phi$ is a quantifier-free formula in the theory of arithmetic over integers, and $\Psi$ and $\Xi$ are quantifier-free formulas in the combined theory of arrays and arithmetic over integers.

Static single assignment (SSA) [27] is a well-known technique for renaming scalar variables such that a variable is written at most once in a program. For our purposes, we also wish to rename arrays so that each loop updates its own version of an array and multiple writes to an array element within the same loop happen on different versions of the array. Array SSA [20] renaming has been studied earlier in the context of compilers to achieve this goal. We propose using SSA renaming for both scalars and arrays as a pre-processing step of our analysis. Therefore, we assume henceforth that the input program is SSA renamed (for both scalars and arrays). We also assume that the post-condition is expressed in terms of these SSA renamed scalar and array variables.

We represent a program using a *control flow graph* $G = (Locs, Edges, \mu)$, where $Locs$ denotes the set of control locations (nodes) of the program, $Edges \subseteq Locs \times Locs \times \{\mathbf{tt}, \mathbf{ff}, \mathsf{U}\}$ represents the flow of control and $\mu : Locs \rightarrow \mathsf{AssignSt} \cup \mathsf{BoolE}$ annotates every node in $Locs$ with either an assignment statement (of the form $v := \mathsf{E}$ or $A[\mathsf{E}] := \mathsf{E}$) from the set of assignment statements $\mathsf{AssignSt}$, or a Boolean condition. Two distinguished control locations, called $\mathsf{Start}$ and $\mathsf{End}$ in $Locs$, represent the entry and exit points of the program. An edge $(n_1, n_2, label)$ represents flow of control from $n_1$ to $n_2$ without any other intervening node. It is labeled $\mathbf{tt}$ or $\mathbf{ff}$ if $\mu(n_1)$ is a Boolean condition, and is labeled $\mathsf{U}$ otherwise. If $\mu(n_1)$ is a Boolean condition, there are two outgoing edges from $n_1$, labeled $\mathbf{tt}$ and $\mathbf{ff}$ respectively, and control flows from $n_1$ to $n_2$ along $(n_1, n_2, label)$ only if $\mu(n_1)$ evaluates to *label*. If $\mu(n_1)$ is an assignment statement, there is a single outgoing edge from $n_1$, and it is labeled $\mathsf{U}$. Henceforth, we use CFG to refer to the control flow graph.

A CFG may have cycles due to the presence of loops in the program. A *back-edge* of a loop is an edge from the node corresponding to the last statement in the loop body to the node representing the loop head. An *exit-edge* is an edge from the loop head to a node outside the loop body. An *incoming-edge* is an edge to the loop head from a node outside the loop body. We assume that every loop has exactly one *back-edge*, one *incoming-edge* and one *exit-edge*. For technical reasons, and without loss of generality, we also assume that the *exit-edge* of a loop always goes to a "nop" node (say, having a statement `x = x;`).

Given a program, the program dependence graph (or PDG) $G = (V, DE, CE)$ represents data and control dependencies among program statements. Here, $V$ denotes vertices representing assignment statements and boolean expressions, $DE \subseteq V \times V$ denotes data dependence edges and $CE \subseteq V \times V$ denotes control dependence edges. Standard dataflow analysis identifies dependencies between program variables and thereby among statements. Dependence between statements updating array elements requires a more careful analysis. Let $S_1$ and $S_2$ be two statements in loops $L_1$ and $L_2$ where there is a control-flow path from $S_1$ to $S_2$ in the CFG. Suppose $S_1$ is of the form $A[f(i_1, N)] = F(\ldots)$; where $f$ is an array index expression, $i_1$ is the loop counter variable of $L_1$, and $F$ is an arbitrary expression. Suppose $S_2$ is of the form $X = G(A[g(i_2, N)])$;, where $X$ is a variable or array element, $G$ is an arbitrary expression, and $g$ is an array index expression.

**Definition 1.** *We say that* $S_2$ *in* $L_2$ ***depends*** *on* $S_1$ *in* $L_1$ *if there exists* $i_1, i_2$ *such that* $0 \leq i_1 < k_{L_1}(N)$ *and* $0 \leq i_2 < k_{L_2}(N)$ *and* $f(i_1, N) = g(i_2, N)$*.*

The routine ComputeRefinedPDG shown in Algorithm 1 constructs and refines the program dependence graph $G = (V, DE, CE)$ for the input program $\mathsf{P}_N$. It uses the function ConstructPDG (line 1) based on the technique of [11] to create an initial graph. For a node $n$ in $G$, let $def(n)$ and $uses(n)$ refer to the set of variables/array elements defined and used, respectively, in the statement/boolean expression corresponding to $n$. Similarly, let $subscript(v, n)$ refer to the index expression of the array element $v$ referred to at node $n$. Predicate $is\text{-}array(v)$ evaluates to true if $v$ is an array element and false if $v$ is a

**Algorithm 1** COMPUTEREFINEDPDG($\mathsf{P}_N$ : Program)

1: $G(V, DE, CE)$ := CONSTRUCTPDG($\mathsf{P}_N$);
2: **if** $\exists v, n, n'.\ (n, n') \in DE \wedge \textit{is-array}(v) \wedge v \in \textit{def}(n) \wedge v \in \textit{uses}(n')$ **then**
3:     **if** $n$ is part of a loop $L$ **then**
4:         $\ell$ := loop counter of $L$;
5:         Let $\phi(n)$ be the constraint $(0 \leq \ell < k_L)$;
6:     **else**
7:         Let $\phi(n)$ be *true*;
8:     **if** $n'$ is part of a loop $L'$ **then**
9:         $\ell'$ := loop counter of $L'$;
10:         Let $\phi'(n')$ be the constraint $(0 \leq \ell' < k_{L'})$;
11:     **else**
12:         Let $\phi'(n')$ be *true*;
13:     **if** $\phi(n) \wedge \phi(n') \wedge \big(\textit{subscript}(v, n) = \textit{subscript}(v, n')\big)$ is unsatisfiable **then**
14:         $DE = DE \setminus \{(n, n')\}$; ▷ Remove dependence edges with non-overlapping subscripts
15: **return** $G(V, DE, CE)$;

**Algorithm 2** PEELALLLOOPS($(\textit{Locs}, \textit{Edges}, \mu)$ : CFG of $\mathsf{P}_N$)

1: $\mathsf{P}^p_N := (\textit{Locs}^p, \textit{Edges}^p, \mu^p)$, where $L^p = \textit{Locs}$, $\textit{Edges}^p = \textit{Edges}$, $\mu^p = \mu$; ▷ Copy of $\mathsf{P}_N$
2: $\textit{peelNodes} := \varnothing$;
3: **for** each loop $L \in$ LOOPS($\mathsf{P}^p_N$) **do**
4:     Let $k_L(N)$ be the expression for iteration count of $L$ in $\mathsf{P}^p_N$;
5:     $\textit{peelCount}$ := SIMPLIFY($k_L(N) - k_L(N-1)$);
6:     **if** $\textit{peelCount}$ is non-constant **then throw** "Failed to peel non-constant number of iterations";
7:     $\langle \mathsf{P}^p_N, \textit{Locs}' \rangle$ := PEELSINGLELOOP($\mathsf{P}^p_N, L, k_L(N-1), \textit{peelCount}$);
    ▷ Transforms loop $L$ so that last $\textit{peelCount}$ iterations of $L$ are peeled/unrolled. Updated CFG and newly created CFG nodes for the peeled iterations are returned by PEELSINGLELOOP.
8:     $\textit{peelNodes} := \textit{peelNodes} \cup \textit{Locs}'$;
9: **return** $\langle \mathsf{P}^p_N, \textit{peelNodes} \rangle$; scalar variable. Note that lines 2-14 of COMPUTEREFINEDPDG removes data dependence edges between nodes of $G$ that do not satisfy Definition 1.

### 3.2 Core Modules in the Technique

***Peeling the Loops.*** To relate $\mathsf{P}_N$ to $\mathsf{P}_{N-1}$, we first ensure that the corresponding loops in both programs iterate the same number of times by *peeling* extra iterations from the loops in $\mathsf{P}_N$. This is done by routine PEELALLLOOPS shown in Algorithm 2. The algorithm first makes a copy, viz. $\mathsf{P}^p_N$, of the input CFG $\mathsf{P}_N$. Let LOOPS($\mathsf{P}^p_N$) denote the set of loops of $\mathsf{P}^p_N$, and let $k_L(N)$ and $k_L(N-1)$ denote the number of times loop $L$ iterates in $\mathsf{P}^p_N$ and $\mathsf{P}^p_{N-1}$ respectively. The difference $k_L(N) - k_L(N-1)$, computed in line 5, gives the extra iterations of loop $L$ in $\mathsf{P}^p_N$. If this difference is not a constant, we currently report a failure of our technique (line 6). Otherwise, routine PEELSINGLELOOP transforms loop $L$ of $\mathsf{P}^p_N$ as follows: it replaces the termination condition $(\ell < k_L(N))$ of $L$ by $(\ell < k_L(N-1))$. It also peels (or unrolls) the last $(k_L(N) - k_L(N-1))$ iterations of $L$ and adds control flow edges such that the the peeled iterations are executed immediately after the loop body is iterated $k_L(N-1)$ times. Effectively, PEELSINGLELOOP unrolls/peels the last $(k_L(N) - k_L(N-1))$ iterations of loop $L$ in $\mathsf{P}^p_N$. The transformed CFG is returned as the updated $\mathsf{P}^p_N$ in line 7. In addition, PEELSINGLELOOP also returns the set $\textit{Locs}'$ of all CFG nodes newly added while peeling the loop $L$. The overall updated CFG and the set of all peeled nodes obtained after peeling all loops in $\mathsf{P}^p_N$ is returned in line 9.

**Lemma 1.** *$\{\varphi_N\}$ $\mathsf{P}_N$ $\{\psi_N\}$ holds iff $\{\varphi_N\}$ $\mathsf{P}^p_N$ $\{\psi_N\}$ holds.*

**Algorithm 3** ComputeAffected($\mathsf{P}_N$ : Program, *peelNodes* : Peeled Statements)

1: $G(V, DE, CE)$ := ComputeRefinedPDG($\mathsf{P}_N$);
2: AffectedVars := $\{N\}$; ▷ $N$ is in the affected set
3: **repeat**
4: WorkList := $V \setminus peelNodes$; ▷ all non-peeled nodes in $G$
5: **while** WorkList $\neq \{\}$ **do**
6: Remove a node $n$ from WorkList;
7: **if** $\exists v.\ is\text{-}array(v) \wedge (\exists u.\ u \in subscript(v, n) \wedge u \in$ AffectedVars) **then**
8: AffectedVars := AffectedVars $\cup\ v$;
9: **if** $\exists v.\ v \in uses(n)$ **then**
10: **if** $\exists m.\ m \in reaching\text{-}def(v, n) \wedge m \in peelNodes$ **then**
11: AffectedVars := AffectedVars $\cup\ def(n)$;
12: **if** $\exists m.\ m \in reaching\text{-}def(v, n) \wedge def(m) \in$ AffectedVars **then**
13: AffectedVars := AffectedVars $\cup\ def(n)$;
14: **if** $v \in$ AffectedVars $\wedge\ n$ is a assignment node **then**
15: AffectedVars := AffectedVars $\cup\ def(n)$;
16: **if** $v \in$ AffectedVars $\wedge\ n$ is a predicate node **then**
17: **for** each edge $(n, n') \in CE$ **do**
18: AffectedVars := AffectedVars $\cup\ def(n')$;
19: **until** AffectedVars does not change
20: **return** AffectedVars;

***Affected Variable Analysis.*** Before we discuss the generation of $\partial\mathsf{P}_N$, we present an analysis that identifies variables/array elements that may take different values in $\mathsf{P}_N$ and $\mathsf{P}_{N-1}$. For example, the first $k_L(N-1)$ iterations of $L$ in $\mathsf{P}_N$ may not be semantically equivalent to the (entire) $k_L(N-1)$ iterations of $L$ in $\mathsf{P}_{N-1}$. This is because the semantics of statements in $L$ may depend on the value of $N$ either directly or indirectly. We call variables/array elements updated in such statements as *affected* variables. For every loop with statements having potentially different semantics in $\mathsf{P}_N$ and $\mathsf{P}_{N-1}$, the difference program $\partial\mathsf{P}_N$ must have a version of the loop with statements that restore the effect of the first $k_L(N-1)$ iterations of $L$ in $\mathsf{P}_N$ after the (entire) $k_L(N-1)$ iterations of $L$ in $\mathsf{P}_{N-1}$ have been executed. Furthermore, for statements in $\mathsf{P}_N$ that are not enclosed within loops but have potentially different semantics from the corresponding statements in $\mathsf{P}_{N-1}$, $\partial\mathsf{P}_N$ must also rectify the values of variables/array elements updated in such statements.

Subroutine ComputeAffected, shown in Algorithm 3, computes the set of *affected* variables $\mathsf{P}_N$. We first construct the program dependence graph by calling the function ComputeRefinedPDG (line 1) defined in Algorithm 1. Let AffectedVars represent the set of *affected* variables/array elements. We initialize it (line 2) with variable $N$ since its value is different in $\mathsf{P}_N$ and $\mathsf{P}_{N-1}$. For a node $n$ in the PDG $G$, we use *reaching-def*$(v, n)$ to refer to the set of nodes where the variable/array element $v$ is defined and the definition reaches its use at node $n$. In line 4, we collect nodes in the graph that are not the ones peeled from loops in $\mathsf{P}_N$. The loop in lines 5-18 iterates over the collected nodes to identify affected variables. If a variable in the index expression of an array access is affected then that array element is considered affected (lines 7-8). A definition at a node $n$ is affected (marked in line 11) if any variable $v$ used in the statement (checked in line 9) is defined in a *peeled* node (line 10). Similarly if the reaching definition of $v$ is affected (line 12) the definition at $n$ is affected (line 13). A variable defined in terms of an affected variable is also deemed to be affected (lines 14-

15). Finally, a variable definition that is control dependent on an affected variable is also considered affected (lines 16-18). The computation of affected variables is iterated until the set AffectedVars saturates.

**Lemma 2.** *Variables/Array elements not present in* AffectedVars *have the same value after* $k_L(N-1)$ *iterations of its enclosing loop (if any) in* $P_{N-1}$ *as in* $P_N$*.*

***Generating the Difference Program*** $\partial \mathsf{P}_\mathbf{N}$***.*** The routine ProgramDiff in Algorithm 4 shows how the difference program is computed. We peel each loop in the program and collect the list of peeled nodes (line 1) using Algorithm 2. We then compute the set of *affected* variables (line 2) using Algorithm 3. The difference program $\partial \mathsf{P}_N$ inherits the skeletal structure of the program $\mathsf{P}_N$ after peeling each loop (line 4). The algorithm then traverses the CFG of each loop in $\mathsf{P}_N$ and removes the loops (lines 16-17) that do not update any *affected* variables from $\partial \mathsf{P}_N$. For every CFG node in other loops, it determines the corresponding node type (assignment or branch) and acts accordingly (lines 7-14). To explain the intuition behind the steps of this algorithm, we use the convention that all variables and arrays of $\mathsf{P}_{N-1}$ have the suffix `_Nm1` (for N-minus-1), while those of $\mathsf{P}_N$ have the suffix `_N`. This allows us to express variables/array elements of $\mathsf{P}_N$ in terms of the corresponding variables/array elements of $\mathsf{P}_{N-1}$ in a systematic way in $\partial \mathsf{P}_N$, given that the intended composition is $\mathsf{P}_{N-1}; \partial \mathsf{P}_N$.

For assignment statements using simple arithmetic operators (`+,-,*,/`), the sub-routine AssignmentDiff in Algorithm 4 computes a "difference" statement as follows. We assume that Nodes($L$) returns the set of CFG nodes in loop $L$. For every assignment statement of the form `v = E;` in $L$, a corresponding statement is generated in $\partial \mathsf{P}_N$ that expresses `v_N` in terms of `v_Nm1` and the difference (or ratio) between versions of variables/arrays that appear as sub-expressions in `E` in $\mathsf{P}_{N-1}$ and $\mathsf{P}_N$. For example, the statement `A_N[i] = B_N[i] + v_N;` in $\mathsf{P}_N$ gives rise to the "difference" statement `A_N[i] = A_Nm1[i] + (B_N[i] - B_Nm1[i]) + (v_N - v_Nm1);` in $\partial \mathsf{P}_N$. Similarly, the statement `A_N[i] = B_N[i] * v_N;` in $\mathsf{P}_N$ gives rise to the "difference" statement `A_N[i] = A_Nm1[i] * (B_N[i] / B_Nm1[i]) * (v_N / v_Nm1);` under the assumption `B_Nm1[i] * v_Nm1` $\neq 0$.

There are additional kinds of statements that need special processing when generating $\partial \mathsf{P}_N$. These relate to accumulation of differences (or ratios). For example, if $\mathsf{P}_N$ has a loop `for(i = 0; i < N; i++) sum_N = sum_N + A_N[i];` then the difference `A_N[i] - A_Nm1[i]` is aggregated over all indices from 0 through $N-2$. In this case, the corresponding "difference" loop in $\partial \mathsf{P}_N$ has the following form: `sum_N = sum_Nm1; for(i = 0; i < N-1; i++) sum_N = sum_N + (A_N[i] - A_Nm1[i]);`. A similar aggregation for multiplicative ratios can also be defined. Sub-routine AggregateAssignmentDiff in Algorithm 4 generates these "difference" statements.

Note that expressions like `(B_N[i] - B_Nm1[i])` or `(v_N/v_Nm1)` can often be simplified from the already generated part of $\partial \mathsf{P}_N$. For example, if the already generated part has a statement of the form `B_N[i] = B_Nm1[i] + expr1;` or `v_N = expr2*v_Nm1;`, and if `expr1` and `expr2` are constants or functions of $N$ and loop counters, then we can use `expr1` for `B_N[i] - B_Nm1[i]` and `expr2` for

**Algorithm 4** PROGRAMDIFF($\mathsf{P}_N$: program)

$\langle \mathsf{P}_N, peelNodes\rangle := \text{PEELALLLOOPS}(\mathsf{P}_N)$;
$\mathsf{AffectedVars} := \text{COMPUTEAFFECTED}(\mathsf{P}_N, peelNodes)$;
Let the CFG of $\mathsf{P}_N$ be $(Locs, E, \mu)$;
$\partial\mathsf{P}_N := (Locs', E', \mu')$, where $Locs' := Locs$, $E' := E$, and $\mu' := \emptyset$;
**for** each loop $L \in \text{LOOPS}(\mathsf{P}_N)$ **do**
    **if** $\exists v$ such that $v$ is updated in $L$ and $v \in \mathsf{AffectedVars}$ **then**
        **for** each node $n \in \text{NODES}(L)$ **do**
            $st_N := \mu(n)$;
            **if** $st_N$ is of the form $w_N := r_N^1 \texttt{ op } r_N^2$ **then**
                $\mu'(n) := \text{ASSIGNMENTDIFF}(\ w_N := r_N^1 \texttt{ op } r_N^2\ )$;
            **else if** $st_N$ is of the form $w_N := w_N \texttt{ op } r_N^1$ wherein $w_N$ is a scalar **then**
                $\mu'(n) := \text{AGGREGATEASSIGNMENTDIFF}(\ L,\ w_N := w_N \texttt{ op } r_N^1\ )$;
            **else** ▷ $st_N$ is a conditional statement
                $\mu'(n) := \text{BRANCHDIFF}(\ st_N,\ \mathsf{AffectedVars}\ )$;
    **else** ▷ Remove loop $L$ from CFG of $\partial\mathsf{P}_N$
        $(n_1, n, \mathsf{U}) := \text{INCOMINGEDGE}(L)$; $(n, n_2, \mathbf{ff}) := \text{EXITEDGE}(L)$;
        $E' := E' \setminus \{(n_1, n, \mathsf{U}), (n, n_2, \mathbf{ff})\} \cup \{(n_1, n_2, \mathsf{U})\}$; $Locs' := Locs' \setminus \mathsf{Nodes}(L)$;
**return** $\partial\mathsf{P}_N$;

ASSIGNMENTDIFF( $w_N := r_N^1 \texttt{ op } r_N^2$ )

Let `invop` be the arithmetic inverse operator of `op`;
    ▷ `+ and − are inverse operators of each other, and so are × and ÷`
**if** $\texttt{op} \in \{+, \times\}$ **then**
    **return** $w_N := w_{Nm1} \texttt{ op } (\text{SIMPLIFY}(r_N^1 \texttt{ invop } r_{Nm1}^1) \texttt{ op } \text{SIMPLIFY}(r_N^2 \texttt{ invop } r_{Nm1}^2))$;
**else if** $\texttt{op} \in \{-, \div\}$ **then**
    **return** $w_N := w_{Nm1} \texttt{ invop } (\text{SIMPLIFY}(r_N^1 \texttt{ op } r_{Nm1}^1) \texttt{ op } \text{SIMPLIFY}(r_N^2 \texttt{ op } r_{Nm1}^2))$;
**else**
    **throw** "Specified operator not handled";

AGGREGATEASSIGNMENTDIFF( $L$: loop, $w_N := w_N \texttt{ op } r_N^1$ )

$n_{fresh} := \text{FRESHNODE}()$; $\mu'(n_{fresh}) := (w_N := w_{Nm1})$; $Locs' := Locs' \cup \{n_{fresh}\}$;
$(n', n'', \mathsf{U}) := \text{INCOMINGEDGE}(L)$;
$E' := E' \setminus \{(n', n'', \mathsf{U})\} \cup \{(n', n_{fresh}, \mathsf{U}), (n_{fresh}, n'', \mathsf{U})\}$;
**if** $\texttt{op} \in \{+, *\}$ **then**
    **return** $w_N := w_N \texttt{ op } \text{SIMPLIFY}(r_N^1 \texttt{ invop } r_{Nm1}^1)$;
**else if** $\texttt{op} \in \{-, \div\}$ **then**
    **return** $w_N := w_N \texttt{ op } \text{SIMPLIFY}(r_N^1 \texttt{ op } r_{Nm1}^1)$;
**else**
    **throw** "Specified operator not handled";

BRANCHDIFF( $st_N$: branch condition, $\mathsf{AffectedVars}$ : set of affected variables )

Let $n$ be CFG node corresponding to $st_N$;
**if** ($\exists v$ such that v is read in $st_N$ and $v \in \mathsf{AffectedVars}$) $\vee$ ($st_N \neq st_{N-1}$ is satisfiable) **then**
    **throw** "Branch conditions in $\mathsf{P}_N$ and $\mathsf{P}_{N-1}$ may not evaluate to same value";
**else**
    **return** $st_{N-1}$;

`v_N/v_Nm1` respectively. We use these optimizations aggressively in the function SIMPLIFY used in ASSIGNMENTDIFF and AGGREGATEASSIGNMENTDIFF.

For every CFG node representing a conditional branch in $\mathsf{P}_N$, Algorithm BRANCHDIFF is used to determine if the result of the condition check can differ in $\mathsf{P}_N$ and $\mathsf{P}_{N-1}$. If not, the conditional statement can be retained as such in the "difference" program. Otherwise, our current technique cannot compute $\partial\mathsf{P}_N$ and we report a failure of our technique (see body of BRANCHDIFF). For example, the conditional statement `if (t3 == 0)` in line 10 of Fig. 1(a) behaves identically in $\mathsf{P}_{N-1}$ and $\mathsf{P}_N$, and therefore can be used as is in the loop in the difference program.

**Lemma 3.** *$\partial\mathsf{P}_N$ generated by* PROGRAMDIFF *is such that, for all $N > 1$, $\{\varphi(N)\}\ \mathsf{P}_{N-1}; \partial\mathsf{P}_N\ \{\psi(N)\}$ holds iff $\{\varphi(N)\}\ \mathsf{P}_N\ \{\psi(N)\}$ holds.*

**Algorithm 5** SimplifyDiff($\partial\mathsf{P}_N$: difference program)

$\partial\mathsf{P}_N := (Locs, E, \mu)$
$\partial\mathsf{P}'_N := (Locs', E', \mu')$, where $Locs' := Locs$, $E' := E$, and $\mu' := \mu$;
**for** each loop $L \in$ Loops($\partial\mathsf{P}_N$) **do**
  $(n_1, n, \mathsf{U}) :=$ IncomingEdge($L$); $(n, n_2, \mathbf{ff}) :=$ ExitEdge($L$);
  **if** Loop body of $L$ is of the form $w_N := w_N$ op $expr$, wherein $w_N$ is a scalar variable **then**
    $n_{acc} =$ FreshNode();
    **if** op $\in \{+, -\}$ **then**
      $\mu'(n_{acc}) := (w_N := w_N$ op Simplify$(k_L(N-1) * expr))$;
    **else if** op $\in \{*, \div\}$ **then**
      $\mu'(n_{acc}) := (w_N := w_N$ op Simplify$(expr^{k_L(N-1)}))$;
    **else throw** "Specified operator not handled";
    $E' := E'$ - $\{(n_1, n, \mathsf{U}), (n, n_2, \mathbf{ff})\} \cup \{(n_1, n_{acc}, \mathsf{U}), (n_{acc}, n_2, \mathsf{U})\}$;
    $Locs' := Locs' -$ Nodes$(L) \cup \{n_{acc}\}$ ;
  **if** Loop body of $L$ is of the form $w_N := w_{Nm1}$ or $w_N := w_N$ **then**
    $E' := E' - \{(n_1, n, \mathsf{U}), (n, n_2, \mathbf{ff})\} \cup \{(n_1, n_2, \mathsf{U})\}$; $Locs' := Locs' -$ Nodes$(L)$;
**return** $\partial\mathsf{P}'_N$

***Simplifying the Difference Program.*** While we have described a simple strategy to generate $\partial\mathsf{P}_N$ above, this may lead to redundant statements in the naively generated "difference" code. For example, we may have a loop like `for (i=0; i < N-1; i++)  A_N[i] = A_Nm1[i];`. Our implementation aggressively optimizes and removes such redundant code, renaming variables/arrays as needed (see routine SimplifyDiff in Algorithm 5). The program $\partial\mathsf{P}_N$ may also contain loops that compute values of variables that can be accelerated. For example, we may have a loop `for(i=0; i < N-1; i++)  sum = sum + 1;`. Algorithm SimplifyDiff removes this loop and introduces the statement `sum = sum + (N-1);`. This helps in $\partial\mathsf{P}_N$ having fewer and simpler loops in a lot of cases.

**Lemma 4.** *Program $\partial\mathsf{P}'_N$ generated by* SimplifyDiff *is such that, for all $N > 1$, $\{\varphi(N)\}\ \mathsf{P}_{N-1};\partial\mathsf{P}'_N\ \{\psi(N)\}$ holds iff $\{\varphi(N)\}\ \mathsf{P}_{N-1};\partial\mathsf{P}_N\ \{\psi(N)\}$ holds.*

***Generating the Difference Pre-condition $\partial\varphi(\mathbf{N})$.*** We now present a simple syntactic algorithm, called SyntacticDiff, for generation of the difference pre-condition $\partial\varphi(N)$. Although this suffices for all our experiments, for the sake of completeness, we present later a more sophisticated algorithm for generating $\partial\varphi(N)$ simultaneously with $\mathsf{Pre}(N)$.

Formally, given $\varphi(N)$, algorithm SyntacticDiff generates a formula $\partial\varphi(N)$ such that $\varphi(N) \rightarrow (\varphi(N-1) \wedge \partial\varphi(N))$. Observe that if such a $\partial\varphi(N)$ exists, then $\varphi(N) \rightarrow \varphi(N-1)$ holds as well. Therefore, we can use the validity of $\varphi(N) \rightarrow \varphi(N-1)$ as a test to decide the existence of $\partial\varphi(N)$.

If $\varphi(N)$ is of the syntactic form $\forall i \in \{0 \ldots N\}\ \widehat{\varphi}(i)$, then $\partial\varphi(N)$ is easily seen to be $\hat{\varphi}(N)$. If $\varphi(N)$ is of the syntactic form $\varphi^1(N) \wedge \cdots \wedge \varphi^k(N)$, then $\partial\varphi(N)$ can be computed as $\partial\varphi^1(N) \wedge \cdots \wedge \partial\varphi^k(N)$. Finally, if $\varphi(N)$ doesn't belong to any of these syntactic forms or if condition 2(a) of Theorem 1 is violated by the heuristically computed $\partial\varphi(N)$, then we over-approximate $\partial\varphi_N$ by $\mathsf{True}$. For a large fraction of our benchmarks, the pre-condition $\varphi(N)$ was $\mathsf{True}$, and hence $\partial\varphi(N)$ was also $\mathsf{True}$.

***Generating the Formula $\mathsf{Pre}(\mathbf{N-1})$.*** We use Dijsktra's weakest pre-condition computation to obtain $\mathsf{Pre}(N-1)$ after the "difference" pre-condition $\partial\varphi(N)$ and the "difference" program $\partial\mathsf{P}_N$ have been generated. The weakest pre-condition

**Algorithm 6** FPIVerify($\mathsf{P}_N$: program, $\varphi(N)$: pre-condn, $\psi(N)$: post-condn)

1: **if** Base case check $\{\varphi(1)\}$ $\mathsf{P}_1$ $\{\psi(1)\}$ fails **then**
2: **return** "Counterexample found!";
3: $\partial\varphi(N)$ := SyntacticDiff($\varphi(N)$);
4: $\partial\mathsf{P}_N$ := ProgramDiff($\mathsf{P}_N$);
5: $\partial\mathsf{P}_N$ := SimplifyDiff($\partial\mathsf{P}_N$); ▷ Simplify and Accelerate loops
6: $i := 0$;
7: $\mathsf{Pre}_i(N) := \psi(N)$;
8: $c\_\mathsf{Pre}_i(N) := \mathsf{True}$; ▷ Cumulative conjoined pre-condition
9: **do**
10: **if** $\{c\_\mathsf{Pre}_i(N-1) \wedge \psi(N-1) \wedge \partial\varphi(N)\}$ $\partial\mathsf{P}_N$ $\{c\_\mathsf{Pre}_i(N) \wedge \psi(N)\}$ **then**
11: **return** True; ▷ Assertion verified
12: $i := i + 1$;
13: $\mathsf{Pre}_i(N-1)$ := LoopFreeWP($\mathsf{Pre}_{i-1}(N), \partial\mathsf{P}_N$); ▷ Dijkstra's WP sans WP-for-loops
14: **if** no new $\mathsf{Pre}_i(N-1)$ obtained **then** ▷ Can happen if $\partial\mathsf{P}_N$ has a loop
15: **return** FPIVerify($\partial\mathsf{P}_N$, $c\_\mathsf{Pre}_{i-1}(N-1) \wedge \psi(N-1) \wedge \partial\varphi(N)$, $c\_\mathsf{Pre}_{i-1}(N) \wedge \psi(N)$);
16: **else**
17: $c\_\mathsf{Pre}_i(N) := c\_\mathsf{Pre}_{i-1}(N) \wedge \mathsf{Pre}_i(N)$;
18: **while** Base case check $\{\varphi(1)\}$ $\mathsf{P}_1$ $\{c\_\mathsf{Pre}_i(1)\}$ passes;
19: **return** False; ▷ Failed to prove by full-program induction can always be computed using quantifier elimination engines in state-of-the-art SMT solvers like Z3 if $\partial\mathsf{P}_N$ is loop-free. In such cases, we use a set of heuristics to simplify the calculation of the weakest pre-condition before harnessing the power of the quantifier elimination engine. If $\partial\mathsf{P}_N$ contains a loop, it may still be possible to obtain the weakest pre-condition if the loop doesn't affect the post-condition. Otherwise, we compute as much of the weakest pre-condition as can be computed from the non-loopy parts of $\partial\mathsf{P}_N$, and then try to recursively solve the problem by invoking full-program induction on $\partial\mathsf{P}_N$ with appropriate pre- and post-conditions.

***Verification by Full-program Induction.*** The basic full-program induction algorithm is presented as routine FPIVerify in Algorithm 6. The main steps of this algorithm are: checking conditions 3(a), 3(b) and 3(c) of Theorem 1 (lines 1, 18 and 10), calculating the weakest pre-condition of the relevant part of the post-condition (line 13), and strengthening the pre-condition and post-condition with the weakest pre-condition thus calculated (line 17). Since the weakest pre-condition computed in every iteration of the loop ($Pre_i(N-1)$ in line 13) is conjoined to strengthen the inductive pre-condition ($c\_Pre_i(N)$ in line 17), it suffices to compute the weakest pre-condition of $Pre_{i-1}(N)$ (instead of $c\_Pre_i(N) \wedge \psi(N)$) in line 13. The possibly multiple iterations of strengthening of pre- and post-conditions is effected by the loop in lines 9-18. In case the loop terminates via the `return` statement in line 11, the inductive claim has been successfully proved. If the loop terminates by a violation of the condition in line 18, we report that verification by full-program induction failed. In case $\partial\mathsf{P}_N$ has loops and no further weakest pre-conditions can be generated, we recursively invoke FPIVerify on $\partial\mathsf{P}_N$ in line 15. This situation arises if, for example, we modify the example in Fig. 1(a) by having the statement `C[t3] = N;` (instead of `C[t3] = 0;`) in line 10. In this case, $\partial\mathsf{P}_N$ has a single loop corresponding to the third loop in Fig. 1(a). The difference program of $\partial\mathsf{P}_N$ is, however, loop-free, and hence the recursive invocation of full-program induction on $\partial\mathsf{P}_N$ easily succeeds.

**Algorithm 7** FPIDecomposeVerify( i : integer )

1: **do**
2: $\langle \mathsf{Pre}'_i(N-1), \partial\varphi'_i(N)\rangle$ := NextDecomposition($\mathsf{Pre}_i(N-1)$);
3: Check if (a) $\partial\varphi'_i(N) \wedge \mathsf{Pre}'_i(N-1) \rightarrow \mathsf{Pre}_i(N-1)$,
4: (b) $\varphi(N) \rightarrow \varphi(N-1) \wedge (\partial\varphi'_i(N) \wedge \partial\varphi(N))$,
5: (c) $\mathsf{P}_{N-1}$ does not update any variable or array element in $\partial\varphi'_i(N)$
6: **if** any check in lines 3-5 fails **then**
7: **if** HasNextDecomposition($\mathsf{Pre}_i(N-1)$) **then**
8: **continue**;
9: **else**
10: **return** False;
11: **if** $\{c\_\mathsf{Pre}_{i-1}(N-1) \wedge \psi(N-1) \wedge \mathsf{Pre}_i(N-1) \wedge \partial\varphi(N)\}\ \partial\mathsf{P}_N\ \{c\_\mathsf{Pre}_{i-1}(N) \wedge \psi(N) \wedge \mathsf{Pre}'_i(N)\}$ **then**
12: **return** True; ▷ Assertion verified
13: **else**
14: $c\_\mathsf{Pre}_i(N) := c\_\mathsf{Pre}_{i-1}(N) \wedge \mathsf{Pre}'_i(N)$;
15: $i := i + 1$;
16: $\mathsf{Pre}_i(N-1)$ := LoopFreeWP($\mathsf{Pre}'_{i-1}(N), \partial\mathsf{P}_N$); ▷ Dijkstra's WP sans WP-for-loops
17: **if** $\{\varphi(1)\}\ \mathsf{P}_1\ \{c\_\mathsf{Pre}_{i-1}(1) \wedge \mathsf{Pre}_i(1)\}$ does not hold **then**
18: $i := i - 1$;
19: **else**
20: $prev\_\partial\varphi(N) := \partial\varphi(N)$;
21: $\partial\varphi(N) := \partial\varphi'_{i-1}(N) \wedge \partial\varphi(N)$;
22: **if** FPIDecomposeVerify($i$) returns False **then**
23: $i := i - 1$; $\partial\varphi(N) := prev\_\partial\varphi(N)$;
24: **else**
25: **return** True;
26: **while** HasNextDecomposition($\mathsf{Pre}_i(N-1)$);
27: **return** False;

***Generalized FPI Algorithm.*** While algorithm FPIVerify suffices for all of our experiments, we may not always be so lucky. Specifically, even if $\partial\mathsf{P}_N$ is loop-free, the analysis may exit the loop in lines 9-18 of FPIVerify by violating the base case check in line 18. To handle (at least partly) such cases, we propose the following strategy. Whenever a (weakest) pre-condition $\mathsf{Pre}_i(N-1)$ is generated, instead of using it directly to strengthen the current pre- and post-conditions, we "decompose" it into two formulas $\mathsf{Pre}'_i(N-1)$ and $\partial\varphi'_i(N)$ with a two-fold intent: (a) potentially weaken $\mathsf{Pre}_i(N-1)$ to $\mathsf{Pre}'_i(N-1)$, and (b) potentially strengthen the difference formula $\partial\varphi(N)$ to $\partial\varphi'_i(N) \wedge \partial\varphi(N)$. The checks for these intended usages of $\mathsf{Pre}'_i(N-1)$ and $\partial\varphi'_i(N)$ are implemented in lines 3, 4, 5, 11 and 17 of routine FPIDecomposeVerify, shown as Algorithm 7. This routine is meant to be invoked as FPIDecomposeVerify($i$) after each iteration of the loop in lines 9-18 of routine FPIVerify (so that $\mathsf{Pre}_i(N)$, $c\_\mathsf{Pre}_i(N)$ etc. are initialized properly). In general, several "decompositions" of $\mathsf{Pre}_i(N)$ may be possible, and some of them may work better than others. FPIDecompseVerify permits multiple decompositions to be tried through the use of the NextDecomposition and HasNextDecomposition functions. Lines 22-25 of FPIDecomposeVerify implement a simple back-tracking strategy, allowing a search of the space of decompositions of $\mathsf{Pre}_i(N-1)$. Observe that when we use FPIDecomposeVerify, we simultaneously compute a difference formula $(\partial\varphi'_i(N) \wedge \partial\varphi(N))$ and an inductive pre-condition $(c\_\mathsf{Pre}_{i-1}(N) \wedge \mathsf{Pre}'_i(N))$.

**Lemma 5.** *Algorithms* FPIVerify *and* FPIDecomposeVerify *ensure conditions 2 and 3 of Theorem 1 upon successful termination.*

While we have presented our technique focusing on a single symbolic parameter $N$, a straightforward extension works for multiple independent parameters, multiple independent array sizes, different induction directions, and non-uniform loop termination conditions. For more details, please refer to the long version of our paper at [3].

***Limitations.*** There are several scenarios under which full-program induction may not produce a conclusive result. Currently, we only analyze programs with non-nested loops with $+, -, \times, \div$ expressions in assignments. We also do not handle branch conditions that are dependent on the parameter N (this doesn't include loop conditions, which are handled by unrolling the loop). The technique also remains inconclusive when the difference program $\partial\mathsf{P}_N$ does not have fewer loops than the original program. Reduction in verification complexity of the program, in terms of the number of loops and assignment statements dependent on $N$, is crucial to the success of full-program induction. Finally, our technique may fail to verify a correct program if the heuristics used for weakest pre-condition either fail or return a pre-condition that causes violation of the base case check in line 18 of FPIVerify. Despite these limitations, our experiments show that full-program induction performs remarkably well on a large suite of benchmarks.

## 4 Implementation and Experiments

We have implemented our technique in a prototype tool called Vajra, available at [5]. It takes a C program in SVCOMP format as input. The tool, written in `C++`, is built on top of the LLVM/CLANG [22] 6.0.0 compiler infrastructure and uses Z3 [25] v4.8.7 as the SMT solver to prove Hoare triples for loop-free programs.

We have evaluated Vajra on a test-suite of 42 safe benchmarks inspired from different algebraic functions that compute polynomials as well as a standard array operations such as copy, min, max and compare. Our programs take a symbolic parameter $N$ which specifies the size of each array as well as the number of times each loop executes. Assertions, possibly quantified, are (in-)equalities over array elements, scalars and (non-)linear polynomial terms over $N$.

All experiments were performed on a Ubuntu 18.04 machine with 16GB RAM and running at 2.5 GHz. We have compared Vajra against VIAP(v1.0) [26], VeriAbs(v1.3.10) [8], Booster(v0.2) [1], Vaphor(v1.2) [24] and FreqHorn(v3) [10]. C programs were manually converted to mini-Java as required by Vaphor and CHC's as required by FreqHorn. Our results are shown in Table 1. Vajra verified 36 benchmarks, compared to 23 verified by VIAP, 12 by VeriAbs, 8 by Booster, 5 each by Vaphor and FreqHorn. Vajra was unable to compute the difference program for 5 benchmarks and was inconclusive on 1 benchmark.

Vajra verified 17 benchmarks on which VIAP diverged, primarily due to the inability of VIAP's heuristics to get closed form expressions. VIAP verified 4 benchmarks that could not be verified by the current version of Vajra due to syntactic limiations. Vajra, however, is two orders of magnitude faster than VIAP on programs that were verified by both. Vajra proved 28 benchmarks on which VeriAbs diverged. VeriAbs ran out of time on programs where loop shrinking and merging abstractions were not strong enough

| Name | #L | T1 | T2 | T3 | T4 | T5 | T6 |
|---|---|---|---|---|---|---|---|
| pcomp | 3 | ✓0.68 | TO | TO | ?0.23 | TO | ?0.58 |
| ncomp | 3 | ✓0.68 | TO | TO | ?0.41 | TO | ?0.68 |
| eqnm2 | 2 | ✓0.52 | TO | TO | ?0.07 | TO | ?0.59 |
| eqnm3 | 2 | ✓0.53 | TO | TO | ?0.07 | TO | ?0.56 |
| eqnm4 | 2 | ✓0.51 | TO | TO | ?0.07 | TO | ?0.60 |
| eqnm5 | 2 | ✓0.55 | TO | TO | ?0.07 | TO | ?0.58 |
| sqm | 2 | ✓0.51 | ✓69.7 | TO | ?0.11 | TO | ?0.57 |
| res1 | 4 | ✓0.17 | TO | TO | TO | TO | TO |
| res1o | 4 | ✓0.18 | TO | TO | TO | TO | TO |
| res2 | 6 | ✓0.20 | TO | TO | TO | TO | TO |
| res2o | 6 | ✓0.22 | TO | TO | TO | TO | TO |
| ss1 | 4 | ✓0.40 | TO | TO | ✗0.13 | ?19.2 | ?1.7 |
| ss2 | 6 | ✓0.46 | TO | TO | ✗0.13 | TO | ?9.7 |
| ss3 | 5 | ✓0.35 | TO | TO | ✗0.13 | TO | ?2.1 |
| ss4 | 4 | ✓0.29 | TO | TO | ✗0.13 | TO | ?1.6 |
| ssina | 5 | ✓0.41 | ✓72.5 | TO | TO | TO | ?2.0 |
| sina1 | 2 | ✓0.56 | ✓65.4 | TO | TO | TO | TO |
| sina2 | 3 | ✓0.69 | ✓66.5 | TO | TO | TO | TO |
| sina3 | 4 | ✓0.83 | TO | TO | TO | TO | TO |
| sina4 | 4 | ✓0.85 | TO | TO | TO | TO | TO |
| sina5 | 5 | ✓0.93 | TO | TO | TO | TO | TO |

| Name | #L | T1 | T2 | T3 | T4 | T5 | T6 |
|---|---|---|---|---|---|---|---|
| zerosum1 | 2 | ✓0.33 | ✓62.0 | ✓11 | ✓0.77 | ✗0.29 | TO |
| zerosum2 | 4 | ✓0.46 | ✓75.8 | ✓18 | TO | ✗1.64 | TO |
| zerosum3 | 6 | ✓0.59 | ✓73.1 | ✓39 | TO | ✗3.13 | TO |
| zerosum4 | 8 | ✓0.76 | ✓76.1 | TO | ?18.2 | ✗6.85 | TO |
| zerosum5 | 10 | ✓0.97 | ✓80.6 | TO | ?16.5 | ✗10.4 | TO |
| zerosumm2 | 4 | ✓0.46 | ✓71.5 | ✓24 | TO | ✗1.22 | TO |
| zerosumm3 | 6 | ✓0.59 | ✓70.9 | TO | TO | ✗5.22 | TO |
| zerosumm4 | 8 | ✓0.77 | ✓76.4 | TO | ?16.7 | ✗12.39 | TO |
| zerosumm5 | 10 | ✓0.98 | ✓81.7 | TO | ?18.7 | ✗22.8 | TO |
| zerosumm6 | 12 | ✓1.29 | ✓86.8 | TO | ?16.1 | TO | TO |
| copy9 | 9 | ✓0.69 | ✓86.8 | ✓3.91 | ✓18.8 | TO | ✓0.67 |
| min | 1 | ✓0.48 | ✓23.6 | ✓3.82 | ✓0.52 | ✓0.14 | ✓0.13 |
| max | 1 | ✓0.46 | ✓25.4 | ✓4.70 | ✓1.0 | ✓0.28 | ✓0.18 |
| compare | 1 | ✓0.82 | ✓18.8 | ✓17.9 | ✓0.06 | ✓0.84 | ✓0.31 |
| conda | 3 | ✓0.72 | ✓13.9 | TO | ✓0.07 | ✓0.09 | TO |
| condn | 1 | ?0.51 | ✓14.7 | ✓18.9 | ✓0.02 | ✓0.15 | ✓0.20 |
| condm | 2 | ?0.59 | ✓20.5 | ✓16.7 | ✓0.04 | TO | - |
| condg | 3 | ?0.52 | TO | TO | TO | TO | TO |
| modn | 2 | ?0.63 | ✓22.6 | TO | - | TO | TO |
| mods | 4 | ?0.61 | TO | ✓18.2 | - | - | - |
| modp | 2 | ?0.71 | ✓17.3 | ✓40 | - | ?32 | - |

**Table 1.** First column is the benchmark name. Second column indicates the number loops in the benchmark (excluding the assertion loop). Successive columns indicate the results generated by tools and the time taken where T1 is Vajra, T2 is VIAP, T3 is VeriAbs, T4 is Booster, T5 is Vaphor, T6 is FreqHorn. **✓**indicates assertion safety, **✗**indicates assertion violation, **?** indicates unknown result, and **-** indicates an abrupt stop. All the times are in seconds. TO is time-out of 100 secs.

to prove the assertions. VeriAbs reported 1 program as unsafe due to the imprecision of its abstractions and it proved 4 benchmarks that Vajra could not. Vajra verified 30 benchmarks that Booster could not. Booster reported 4 benchmarks as unsafe due to imprecise abstractions, its fixed-point computation engine reported unknown result on 12 benchmarks and it ended abruptly on 3 benchmarks. Booster also proved 2 benchmarks that couldn't be handled by the current version of Vajra due to syntactic limitations. Vajra verified 32 benchmarks on which Vaphor was inconclusive. Distinguished cell abstraction in Vaphor is unable to prove safety of programs, when the value at each array index needs to be tracked. Vaphor reported 9 programs unsafe due to imprecise abstraction, returned unknown on 2 programs and ended abruptly on 1 program. Vaphor proved a benchmark that Vajra could not. Vajra verified 32 programs on which FreqHorn diverged, especially when constants and terms that appear in the inductive invariant are not syntactically present in the program. FreqHorn ran out of time on 22 programs, reported unknown result on 12 and ended abruptly on 3 benchmarks. FreqHorn verified a benchmark with a single loop that Vajra could not. On an extended set of 231 benchmarks, Vajra verified 110 programs out of 121 safe programs, falsified 108 out of 110 unsafe programs, and was inconclusive on the remaining 13 programs.

## 5 Conclusion

We presented a novel property-driven verification method that performs induction over the entire program via parameter $N$. Significantly, this obviates the need for loop-specific invariants. Experiments show that full-program induction performs remarkably well vis-a-vis state-of-the-art tools for analyzing array manipulating programs. Further improvements in the algorithms for computing difference programs and for strengthening of pre- and post-conditions are envisaged as part of future work.

## Data Availability Statement

The datasets generated and analyzed during the current study are available in the figshare repository: https://doi.org/10.6084/m9.figshare.11875428.v1

## References

[1] Alberti, F., Ghilardi, S., Sharygina, N.: Booster: An acceleration-based verification framework for array programs. In: Proc. of ATVA. pp. 18–23 (2014)

[2] Beyer, D., Henzinger, T.A., Majumdar, R., Rybalchenko, A.: Invariant synthesis for combined theories. In: Proc. of VMCAI. pp. 378–394 (2007)

[3] Chakraborty, S., Gupta, A., Unadkat, D.: Verifying array manipulating programs with full-program induction, https://www.cse.iitb.ac.in/~supratik/publications/papers/FPI_longversion.html

[4] Chakraborty, S., Gupta, A., Unadkat, D.: Verifying Array Manipulating Programs by Tiling. In: Proc. of SAS. pp. 428–449 (2017)

[5] Chakraborty, S., Gupta, A., Unadkat, D.: Verifying Array Manipulating Programs with Full-program Induction - Artifacts TACAS 2020. Figshare (2020). 10.6084/m9.figshare.11875428.v1

[6] Clarke, E., Biere, A., Raimi, R., Zhu, Y.: Bounded model checking using satisfiability solving. FMSD **19**(1), 7–34 (2001)

[7] Cousot, P., Cousot, R., Logozzo, F.: A parametric segmentation functor for fully automatic and scalable array content analysis. In: Proc. of POPL. pp. 105–118 (2011)

[8] Darke, P., Prabhu, S., Chimdyalwar, B., Chauhan, A., Kumar, S., Basakchowdhury, A., Venkatesh, R., Datar, A., Medicherla, R.K.: VeriAbs: Verification by abstraction and test generation. In: TACAS (Competition Contribution). pp. 457–462 (2018)

[9] Ernst, M.D., Perkins, J.H., Guo, P.J., McCamant, S., Pacheco, C., Tschantz, M.S., Xiao, C.: The Daikon system for dynamic detection of likely invariants. Sci. Comput. Program. **69**(1-3), 35–45 (2007)

[10] Fedyukovich, G., Prabhu, S., Madhukar, K., Gupta, A.: Quantified invariants via syntax-guided-synthesis. In: Proc. of CAV. pp. 259–277 (2019)

[11] Ferrante, J., Ottenstein, K.J., Warren, J.D.: The program dependence graph and its use in optimization. TOPLAS **9**(3), 319–349 (1987)

[12] Flanagan, C., Leino, K.R.M.: Houdini, an annotation assistant for ESC/Java. In: Proc. of FME. pp. 500–517 (2001)

[13] Gopan, D., Reps, T.W., Sagiv, S.: A framework for numeric analysis of array operations. In: Proc. of POPL. pp. 338–350 (2005)

[14] Gulwani, S., McCloskey, B., Tiwari, A.: Lifting abstract interpreters to quantified logical domains. In: Proc. of POPL. pp. 235–246 (2008)

[15] Gurfinkel, A., Shoham, S., Vizel, Y.: Quantifiers on demand. In: Proc. of ATVA. pp. 248–266 (2018)

[16] Halbwachs, N., Péron, M.: Discovering properties about arrays in simple programs. In: Proc. of PLDI. pp. 339–348 (2008)

[17] Henzinger, T.A., Hottelier, T., Kovács, L., Rybalchenko, A.: Aligators for arrays (tool paper). In: Proc. of LPAR. pp. 348–356 (2010)

[18] Jacobs, B., Smans, J., Philippaerts, P., Vogels, F., Penninckx, W., Piessens, F.: VeriFast: A powerful, sound, predictable, fast verifier for C and Java. In: Proc. of NFM. pp. 41–55 (2011)

[19] Jhala, R., McMillan, K.L.: Array abstractions from proofs. In: Proc. of CAV. pp. 193–206 (2007)

[20] Knobe, K., Sarkar, V.: Array SSA form and its use in parallelization. In: Proc. of POPL. pp. 107–120 (1998)

[21] Komuravelli, A., Bjorner, N., Gurfinkel, A., McMillan, K.L.: Compositional verification of procedural programs using Horn clauses over integers and arrays. In: Proc. of FMCAD. pp. 89–96 (2015)

[22] Lattner, C.: LLVM and Clang: Next generation compiler technology. In: The BSD Conference. pp. 1–2 (2008)

[23] Liu, J., Rival, X.: Abstraction of arrays based on non contiguous partitions. In: Proc. of VMCAI. pp. 282–299 (2015)

[24] Monniaux, D., Gonnord, L.: Cell Morphing: From array programs to array-free horn clauses. In: Proc. of SAS. pp. 361–382 (2016)

[25] de Moura, L.M., Bjørner, N.: Z3: an efficient SMT solver. In: Proc. ofTACAS. pp. 337–340 (2008)

[26] Rajkhowa, P., Lin, F.: Extending VIAP to handle array programs. In: Proc. ofVSTTE. pp. 38–49 (2018)

[27] Rosen, B.K., Wegman, M.N., Zadeck, F.K.: Global value numbers and redundantcomputations. In: Proc. of POPL. pp. 12–27 (1988)

[28] Seghir, M.N., Brain, M.: Simplifying the verification of quantified arrayassertions via code transformation. In: Proc. of LOPSTR. pp. 194–212(2012)

[29] Sheeran, M., Singh, S., Stålmarck, G.: Checking safety properties usinginduction and a SAT-solver. In: Proc. of FMCAD. pp. 127–144 (2000)

[30] Srivastava, S., Gulwani, S.: Program verification using templates overpredicate abstraction. ACM Sigplan Notices **44**(6), 223–234 (2009)

